# Yeast Rad5 Protein Required for Postreplication Repair Has a DNA Helicase Activity Specific for Replication Fork Regression

**DOI:** 10.1016/j.molcel.2007.07.030

**Published:** 2007-10-12

**Authors:** András Blastyák, Lajos Pintér, Ildiko Unk, Louise Prakash, Satya Prakash, Lajos Haracska

**Affiliations:** 1 Institute of Genetics, Biological Research Center, Hungarian Academy of Sciences, Szeged, Temesvari krt.62, H-6726, Hungary; 2 Department of Biochemistry and Molecular Biology, University of Texas Medical Branch, Galveston, TX 77555-1061, USA

**Keywords:** DNA

## Abstract

Lesions in the template DNA strand block the progression of the replication fork. In the yeast *Saccharomyces cerevisiae,* replication through DNA lesions is mediated by different Rad6-Rad18-dependent means, which include translesion synthesis and a Rad5-dependent postreplicational repair pathway that repairs the discontinuities that form in the DNA synthesized from damaged templates. Although translesion synthesis is well characterized, little is known about the mechanisms that modulate Rad5-dependent postreplicational repair. Here we show that yeast Rad5 has a DNA helicase activity that is specialized for replication fork regression. On model replication fork structures, Rad5 concertedly unwinds and anneals the nascent and the parental strands without exposing extended single-stranded regions. These observations provide insight into the mechanism of postreplicational repair in which Rad5 action promotes template switching for error-free damage bypass.

## Introduction

Unrepaired DNA lesions in the template strand can block the progression of the replication fork. In eukaryotes, the Rad6 and Rad18 proteins play a crucial role in regulating the various lesion bypass processes; Rad6, a ubiquitin-conjugating enzyme, exists in vivo in a tight complex with Rad18, a DNA-binding protein (Bailly et al., 1994, 1997). In the yeast *Saccharomyces cerevisiae*, Rad6-Rad18-mediated ubiquitin conjugation promotes replication through DNA lesions via at least three different pathways. DNA polymerase (Pol)η and Polζ provide means by which synthesis through DNA lesions can be achieved (Johnson et al., 1999; Nelson et al., 1996; Prakash et al., 2005). A third pathway called postreplicational repair (PRR) is dependent upon Rad5 and promotes the repair of discontinuities that form in the DNA synthesized from damaged templates (Torres-Ramos et al., 2002).

Rad5, a member of the SWI/SNF family of ATPases, exhibits a DNA-dependent ATPase activity, and it has a C_3_HC_4_ RING motif characteristic of ubiquitin ligase proteins (Johnson et al., 1992, 1994). In addition to *RAD5,* the *MMS2*, *UBC13*, and *POL30* genes are also required for PRR (Torres-Ramos et al., 1996, 2002). Mms2 forms a specific complex with Ubc13, a ubiquitin-conjugating enzyme, and the Mms2-Ubc13 complex promotes the assembly of polyubiquitin chains linked through lysine 63 of ubiquitin (Hofmann and Pickart, 1999). *POL30* gene encodes the proliferating cell nuclear antigen (PCNA), a sliding clamp on DNA, which plays a key role in the modulation of different lesion bypass processes and provides the central scaffold to which the translesion-synthesis (TLS) polymerases bind (Haracska et al., 2001). In DNA-damaged yeast cells, PCNA becomes monoubiquitinated at lysine 164 by Rad6-Rad18; subsequently, this lysine residue is polyubiquitinated via a lysine 63-linked ubiquitin chain in an Mms2-Ubc13-Rad5-dependent manner (Hoege et al., 2002). In yeast, Rad5 interacts with Rad18 in the Rad6-Rad18 complex, and it physically associates with the Mms2-Ubc13 complex via Ubc13, and this association requires the C_3_HC_4_ motif of Rad5 (Ulrich and Jentsch, 2000). Rad5 presumably promotes PCNA ubiquitination via its role as a ubiquitin ligase by bringing the Mms2-Ubc13 ubiquitin-conjugating activity into close contact with PCNA. Whereas PCNA monoubiquitination activates Polη- and Polζ-dependent TLS, polyubiquitination of the PCNA is required for PRR (Haracska et al., 2004; Hoege et al., 2002). Importantly, the ATPase-defective *rad5* mutant is impaired in PRR to the same degree as is the ubiquitin ligase-defective *rad5* mutant (Gangavarapu et al., 2006). These observations have indicated that both the Rad5 ATPase and ubiquitin ligase activities are indispensable for Rad5 function in PRR. However, the means by which Rad5 ATPase contributes to PRR as well as the molecular mechanism of PRR has remained elusive.

The Rad5-dependent PRR could involve a copy choice type of DNA synthesis, in which the damage is bypassed by template switching using the newly synthesized DNA strand as the template that is formed by replication fork regression (Higgins et al., 1976). Although this model was proposed over 30 years ago, the biochemical activity congruent with the tenets of the model has been lacking for yeast as well as other eukaryotes. The requirement of Rad5 ATPase for PRR, the fact that Rad5 functions in PRR in a nonrecombinational way, and the recent genetic evidence that it promotes error-free replication through DNA lesions by a transient template switching mechanism (Gangavarapu et al., 2006; Liefshitz et al., 1998; Zhang and Lawrence, 2005) are all consistent with a direct role of Rad5 in promoting damage bypass by a copy-choice type of DNA synthesis in which Rad5 could provide the DNA helicase activity necessary for fork regression. However, in previous studies that aimed to examine the possible helicase activity of Rad5, no evidence was found of any DNA unwinding activity in this protein on partial heteroduplex DNAs (Johnson et al., 1994).

Here we examine the role of the ATPase activity of Rad5 in PRR by biochemical means. We provide evidence that Rad5 is a structure-specific DNA helicase that is able to carry out replication fork regression. These observations give strong support for a role of Rad5 in mediating error-free lesion bypass by template switching.

## Results

### Binding and Stimulation of Rad5 ATPase by Various DNA Structures

**Figure 1:**
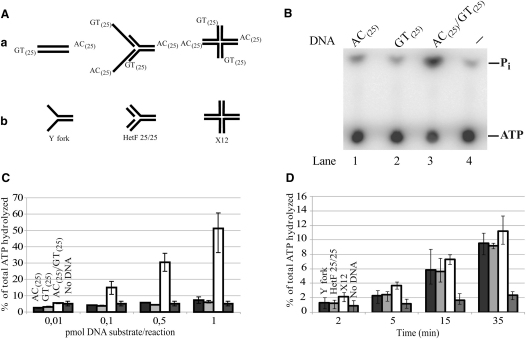
Stimulation of Rad5 ATPase by Various DNA Structures (A) (Aa) Possible DNA structures that can form by the annealing of 50 nucleotide-long, single-stranded oligonucleotides containing 25 dinucleotide repeats of dAdC or dGdT. (Ab) Schematic representation of defined DNA substrates used for Rad5 ATPase assays shown in (D). (B) DNA-dependent ATPase activity of Rad5. Forty nanomolar Rad5 was incubated with 1 mM [γ-^32^P]ATP in the absence or presence of 100 nM single-stranded dAdC_(25)_, single-stranded dGdT_(25)_, or a mixture of these oligonucleotides at 37°C for 30 min. (C) Graphical representation of ATPase activity of Rad5 as the function of DNA concentration. ATPase activity of Rad5 (40 nM) was assayed at various concentrations of DNA (0–100 nM) as described in (B). (D) Kinetics of Rad5 (40 nM) ATPase activity in the presence of Y fork, heterologous fork, and X12 four-way junction-containing DNAs (10 nM). In (C) and (D), the mean of ATPase activity and standard deviation were calculated from three independent experiments.

To address the role of the ATPase activity of Rad5 in PRR, first we examined its DNA dependence. In accord with the previously published data (Johnson et al., 1994), single-stranded phage DNA was stimulatory to Rad5 ATPase activity, but we found that some other single-stranded DNAs (ssDNAs), such as poly-dT, are not (data not shown). Because random sequence ssDNAs can form secondary structures while poly-dT cannot, we suspected that the activation of Rad5 ATPase activity was triggered by secondary structures in DNA rather than by ssDNA. Indeed, the Rad5 ATPase activity was not stimulated by single-stranded 50 nucleotide-long oligomer containing either the dGdT or the dAdC repeats that cannot self-anneal to form secondary structures (Figure 1B, lanes 1 and 2, and Figure 1C). However, a strong stimulation of the ATPase activity occurred with the annealed mixture of dGdT_(25)_ and dAdC_(25)_ oligonucleotides (Figure 1B, lane 3, and Figure 1C). Because the annealing of dGdT_(25)_ and dAdC_(25)_ oligonucleotides can produce, in addition to double-stranded DNA (dsDNA), a variety of other DNA structures, not only partial heteroduplexes but also replication fork-like structures, or even four-way junctions (Figure 1Aa), we also examined whether Rad5 ATPase can be activated by defined replication fork-like DNAs that we constructed from oligonucleotides (Figure 1Ab). Indeed, the ATPase activity of Rad5 was strongly stimulated by Y fork, heterologous three way junction, and four-way junction DNAs (Figure 1D). Next, we tested whether Rad5 in fact can bind these DNA structures by gel shift assays (see Figure S1 in the Supplemental Data available with this article online). In concert with the above-noted DNA dependence of Rad5 ATPase activity, we found that Rad5 was not able to bind to single-stranded DNA, but it bound to partial heteroduplex DNA, Y fork, and more importantly, to three way junction mimicking a replication fork-like structure and a four-way junction, as well (Figure S1).

### Rad5 Has a Replication Fork-Specific DNA Helicase Activity

**Figure 2:**
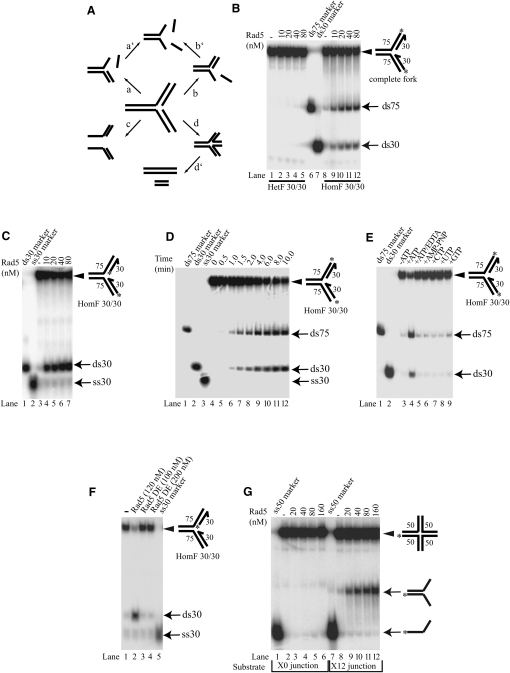
Rad5 Possesses a DNA Helicase Activity Specific for Fork Reversal The DNA substrates in the gel are indicated by arrowheads, while the positions of some of the possible products are shown by arrows. The 3′ ends of oligonucleotides are indicated by half arrows, and the positions of 5′ ^32^P labels are marked with asterisks. (A) Possible outcomes of DNA helicase action on a model replication fork substrate. (B) Replication fork regression activity of Rad5 tested on heterologous fork (HetF) and homologous fork (HomF) model substrates. We note that the formations of double-stranded products indicate fork processing through the d and d′ pathways shown in (A). (C) Homologous replication fork substrate labeled on both nascent strands. (D) Kinetics of Rad5 activity. Processing of homologous fork (HomF 30/30) (0.5 nM) was examined at different times in the presence of 25 nM Rad5. (E) Rad5 helicase activity requires ATP hydrolysis. Cofactor dependence was tested with 5 mM of various ribonucleotides in the presence of 5 mM MgCl_2_, except in lane 5, where 10 mM EDTA was used instead of MgCl_2_. (F) Rad5 ATPase mutant protein has no helicase activity. Rad5 DE refers to mutant Rad5 generated by changing the active-site DE residues at positions 681 and 682 to AA. (G) Rad5 can migrate a moveable four-way junction. The X0 junction is static while the core of the X12 junction is movable, which was flanked with heterologies that are 19 to 20 nucleotides long at the end of each arm.

The spectrum of DNA substrates for binding and stimulation of Rad5 ATPase prompted us to reinvestigate whether Rad5 had a DNA helicase activity on various DNA structures, including replication fork-like substrates. Although there are a number of ways by which a helicase can unwind a model replication fork substrate that could result in a partial heteroduplex, a Y fork, or single-stranded DNAs, a fork regression activity requires that the double-stranded products are formed via a four-way junction intermediate (Figure 2A, see d and d′). Four-way junction can form only on substrates with complementary arms, and therefore the enzyme specific for fork regression must distinguish heterologous and homologous forks. In contrast to a number of structure-specific DNA helicases, such as the Werner, Bloom, and RecQ5beta helicases (Kanagaraj et al., 2006; Mohaghegh et al., 2001), Rad5 was unable to unwind a model replication fork structure containing heterologous arms (Figure 2B, lanes 2–5). We also tested a number of other DNA substrates, including partial heteroduplexes containing either a 5′ or a 3′ single-stranded tail, and heterologous replication fork-like structures where there is either a nascent leading strand or a nascent lagging strand, and found that Rad5 was not able to process these structures (Figure S2). Importantly, however, Rad5 was able to process the model replication fork with homologous arms, as revealed by the appearance of two double-stranded products (Figure 2B, lanes 9–12). Our additional data showed that Rad5 was able to process homologous forks independently of their particular sequence context but did not unwind any heterologous forks (data not shown). When we labeled both the “nascent strand” oligonucleotides, we found that only a double-stranded product consisting of the two annealed nascent strands was formed (Figure 2C). Together with the lack of fork processing activity on heterologous fork, this finding rules out the possibility that Rad5 can unwind one of the nascent strands or one of the template strands independently of the other. Furthermore, kinetic analysis of Rad5 helicase activity confirmed that Rad5 is able to unwind the nascent strands from the two arms and anneal them together as well as anneal the two template strands only in a concerted fashion. As shown in Figure 2D, both double-stranded products were formed with apparently the very same rate and without the appearance of single-stranded intermediates. This provides strong evidence that the outcome of the reaction is not the sum of two or more kinetically separable events: for example, peeling off the nascent strands that can subsequently anneal to form double-stranded products. Thus, Rad5 processes a homologous fork in a highly concerted manner. As expected, ATP hydrolysis was essential for fork processing by Rad5, because it was inhibited by the nonhydrolyzable ATP analog AMP-PNP and also by EDTA, and other ribonucleotides were not able to support the reaction (Figure 2E). Moreover, and importantly, the ATPase-deficient Rad5 DE681,682AA mutant protein (Gangavarapu et al., 2006), in which the active-site DE residues have been changed to alanines, did not promote fork unwinding (Figure 2F).

In order to generate double-stranded products from a model replication fork substrate, Rad5 should also be able to promote the branch migration of four-way junction, which is the first intermediate of fork regression. To examine this directly, we used the conventional synthetic four-way junctions that contained either the nonmovable heterologous arms (X0) or the moveable homologous arms (X12) where a central 12 nucleotide-long homologous core is flanked by heterologous regions 19 to 20 nucleotides long that prevent its spontaneous disruption (McGlynn et al., 2000). Rad5 readily promoted the conversion of the X12 junction into the Y fork, but it was not able to unwind the X0 junction (Figure 2G). The helicase activity of Rad5 was, however, less efficient on the X12 junction than on homologous replication fork substrates (compare Figure 2B with Figure 2G), which suggests that the short sequence heterologies present at the ends of arms in X12 present an obstacle, though not an insurmountable one, for Rad5.

### Rad5 Is Able to Reverse an Asymmetric Fork and Carry Out Extensive Branch Migration

**Figure 3:**
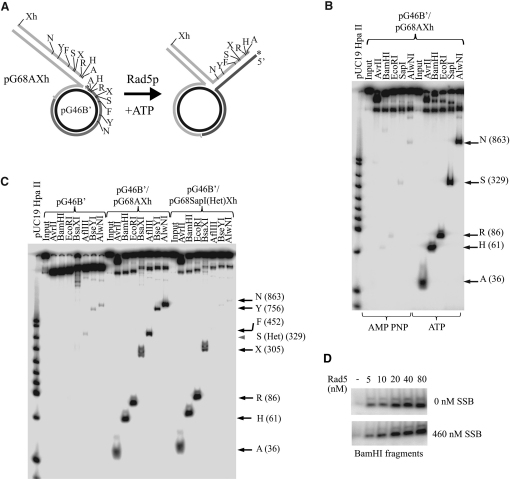
Rad5 Can Regress Plasmid-Sized Model Forks (A) Schematic representation of the joint DNA substrate (pG46B′/pG68AXh) and the outcome of its Rad5-mediated regression. Letters A, H, R, X, S, F, Y, N, and Xh refer to restriction endonuclease sites AvrII, BamHI, EcoRI, BsaXI, SapI, AflIII, BseYI, AlwNI, and XhoI, respectively. The positions of 5′ ^32^P labels on the “lagging strand” are marked with asterisk. (B) The extent of Rad5-dependent fork regression. The restriction enzyme site transfer to the regressed arm by Rad5 was followed in the presence of 5 mM ATP/Mg. The positions of the various restriction products generated by digestion of the regressed fork are indicated. Reaction with 5 mM AMP-PNP/Mg shows the background level of spontaneous regression. (C) Fork regression by Rad5 is progressive. Fork regression by Rad5 was compared on two joint DNA substrates containing either no heterology, or in which a 30 base pair sequence heterology was introduced at the SapI site, shown by arrowhead, of pG68A (named pG68 SapI[Het]). We note that the regression beyond the heterology was blocked, as revealed by the absence of F-, Y-, and N-specific bands. Reaction with gapped DNA (pG46B′) shows background due to nicking at the gap. (D) Fork reversal is not affected by *E. coli* ssDNA-binding protein. Regression through the BamHI site was monitored at various Rad5 concentrations (0–80 nM) in the presence or absence of *E. coli* SSB protein (460 nM).

In vivo, for productive copy-choice synthesis, fork reversal could occur only on asymmetric replication fork that can form upon the uncoupling of leading and lagging strand synthesis and the continuation of lagging strand synthesis that leaves a gap between the two nascent strands (Cordeiro-Stone et al., 1997, 1999; Pages and Fuchs, 2003; Svoboda and Vos, 1995). In addition, based on electron-microscopic measurements, the size of the protruding arm can exceed several hundred base pairs (Higgins et al., 1976). Next, we used a replication fork model substrate that more closely approximates these properties of the stalled replication fork. To generate this substrate, we introduced differently sized complementary gaps in two nearly identical plasmids and, after cleaving one of them and labeling the other, they were annealed to form a joint molecule that mimics a stalled replication fork where the lagging strand is 14 nucleotides longer than the leading strand (Ralf et al., 2006). Regression of this fork can be conveniently monitored by following the movement of the 5′-labeled lagging strand from circular to linear context using restriction enzyme digestion (Figure 3A). We found that Rad5 can readily process this plasmid-sized model substrate in an ATP-dependent manner (Figure 3B). Thus, the “symmetry” of the fork is not a requirement for Rad5 because it is able to regress a fork with a gap between the nascent strands. Remarkably, Rad5 could generate an as long as 863 basepair regressed arm (Figures 3B and 3C), the size that is comparable to the observed extent in vivo (Higgins et al., 1976). To demonstrate progressivity, we tested whether the restriction enzyme sites follow a sequential order in moving into the context of the regressed arm on a substrate in which we introduced a 30 base-pair heterology into the SapI site of the leading arm. As anticipated for a genuine fork reversal reaction, the heterology impeded further regression of the substrate by Rad5 as evidenced by the lack of appearance of downstream enzyme sites in the regressed arm context (Figure 3C).

Formation of the regressed arm without exposing extended ssDNA regions can protect the integrity of the replication fork, and it can also prevent the binding of ssDNA-binding proteins to the nascent strands, which could otherwise inhibit their annealing. To examine this issue, we tested whether *E. coli* ssDNA-binding protein (SSB) inhibits processive fork regression by Rad5. As shown in Figure 3D, we monitored the appearance of the 61 base pair-long arm fragment and found that SSB did not have any inhibitory effect on the Rad5-dependent fork regression, supporting further the theory that ssDNA is not an intermediate product of the reaction.

## Discussion

Although replication through many of the DNA lesions can be accomplished by the action of TLS polymerases, template switching provides for a more general and error-free means of lesion bypass. The biochemical data presented here give strong support for a role of Rad5 in mediating error-free lesion bypass by template switching wherein its DNA helicase activity promotes replication fork regression, a conclusion that is in keeping with genetic observations that have been made with the various *rad5* mutations (Gangavarapu et al., 2006; Johnson et al., 1992; Liefshitz et al., 1998; Torres-Ramos et al., 2002; Zhang and Lawrence, 2005).

Recently, RecQ family members the Werner, Bloom, and RecQ5beta proteins, with well-established role in recombinational repair, have been suggested to perform fork regression in higher-order eukaryotes (Kanagaraj et al., 2006; Machwe et al., 2006; Ralf et al., 2006). However, these RecQ helicases also yield single-stranded DNAs from fork substrates, and importantly, the presence of ssDNA-binding proteins was shown to bias these reactions toward processing forks to structures other than double-stranded products (Kanagaraj et al., 2006). By contrast, Rad5 does not generate single-stranded DNA from fork DNA and is not affected by ssDNA-binding protein; this together with all the other evidence we present here indicates that Rad5 concertedly unwinds and anneals the nascent and the parental strands without exposing extended single-stranded regions. This unique property of Rad5 will ensure that the stalled replication forks are not processed to structures, which are abortive for template switching and the consequent lesion bypass. To our knowledge, Rad5 is the only eukaryotic protein shown to have such an activity, and certainly the only known DNA helicase for which genetic studies have implicated a role in postreplication repair.

**Figure 4:**
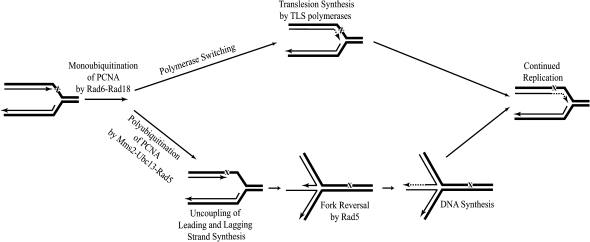
Model for the Role of Rad5 in Lesion Bypass by Template Switching If replication stalls at a DNA lesion, the PCNA monoubiquitination by Rad6-Rad18 enables polymerase switch and lesion bypass by the TLS polymerases to occur. Alternatively, polyubiquitination of PCNA by Mms2-Ubc13-Rad5 promotes PRR. In this model, when replication on the leading strand is blocked by a DNA lesion, lagging strand synthesis continues on. Rad5 then gains access to the asymmetric forks and unwinds both the lagging and leading nascent DNA strands, followed by annealing them as well as annealing the template strands. By this fork regression activity a four-way junction intermediate called “chicken foot” is formed. Following that, a DNA polymerase extends the 3′ OH end of the leading nascent strand by copying from the nascent lagging strand. Finally, the back-migration of the four way junction completes error-free replication through the DNA damage.

In Figure 4, we present a model for Rad6-Rad18-dependent lesion bypass in eukaryotes. If replication stalls upon encountering an unrepaired DNA lesion, PCNA monoubiquitination by Rad6-Rad18 could enable replication through the lesion site by the TLS polymerases. Alternatively, Rad5-mediated template switching could promote lesion bypass. In this case, if the lesion is located on the leading strand template, the leading and the lagging strand polymerases can become uncoupled, and synthesis on the lagging strand can continue way past the blocked nascent leading strand (Cordeiro-Stone et al., 1997, 1999; Pages and Fuchs, 2003; Svoboda and Vos, 1995). Next, the Rad5 helicase action unwinds the nascent strands from their respective templates and then anneals them with one another as well as reanneals the parental strands. The overall outcome of these reactions is the regression of the replication fork to form a four-way junction, similar to a Holliday junction. Following that, the sequences complementary to the damaged region are synthesized on the nascent leading strand using the nascent lagging strand as the template. The reversed fork is then regressed, and synthesis resumes beyond the point of the lesion that completes the error-free damage bypass. We should point out that the proposed model for lesion bypass by Rad5-catalyzed fork reversal is only effective for stalling on the leading strand. Because DNA damage on the leading strand template presents a serious impediment to the progression of the replication fork, whereas lagging strand damage could be bypassed from replication from an adjacent Okazaki fragment and could utilize other means for its bypass, Rad5 would play an indispensable role in rescuing the stalled fork when the lesion is on the leading strand.

The replication fork reversal mediated by Rad5 must be tightly regulated, for otherwise the DNA intermediates generated from fork reversal could provide potential substrates for dangerous recombination events leading to chromosomal rearrangements. One such regulatory circuit is based on PCNA polyubiquitination. Although PRR depends on the polyubiquitination of PCNA by Mms2-Ubc13-Rad5 complex, the precise mechanistic role of PCNA polyubiquitination has remained elusive. We envisage that PCNA polyubiquitination could be important for disrupting the association of some PCNA-bound protein such as the replicative polymerase or another component of the replication ensemble that is otherwise inhibitory to the uncoupling of leading and lagging strand synthesis and for fork reversal.

The replication intermediates that accumulate in yeast cells in response to hydroxyurea (HU) treatment and ultraviolet (UV) exposure have been examined using electron microscopy (EM) and two-dimensional (2D) gel electrophoresis (Sogo et al., 2002; Cotta-Ramusino et al., 2005; Lopes et al., 2006). Although single-stranded gaps accumulate in response to replication blockage in wild-type cells, reversed forks are seen only in *rad53* mutants, where the replication ensemble becomes destabilized. These observations have suggested that the reversed forks that are seen in *rad53* cells represent pathological structures that arise at collapsed replication forks in the absence of Rad53-dependent checkpoint control. We suggest that the fork reversal mediated by Rad5 is a highly regulated and tightly controlled event, and in addition to PCNA polyubiquitination, it likely requires the Rad53-dependent stabilization of the replication fork. We envisage the DNA intermediates of Rad5-mediated fork reversal in vivo to be rather transient, and not subject to processing by nucleases such as the Exo1 processing of reversed forks that occurs in *rad53* cells (Cotta-Ramusino et al., 2005).

Given that the many elements of PRR are conserved from yeast to humans, it is highly probable that the replication fork regression activity of Rad5 has also been conserved during eukaryotic evolution. In this regard, we note that, similar to Rad5, the human SHPRH protein functions as a ubiquitin ligase for Mms2-Ubc13-dependent PCNA polyubiquitination, and similar to Rad5, SHPRH harbors an SWI/SNF2-type helicase domain (Motegi et al., 2006; Unk et al., 2006). Thus, SHPRH too could have the fork regression activity and promote thereby the error-free bypass of DNA lesions by template switching. Interestingly, the SHPRH gene is mutated in a number of cancer cell lines, including those from melanomas and ovarian cancers, which indicates that SHPRH function is an important deterrent to mutagenesis and carcinogenesis in human cells (Sood et al., 2003).

## Experimental Procedures

### Proteins

Wild-type Rad5 and ATPase mutant Rad5 DE681,682AA proteins were purified to apparent homogeneity as described (Gangavarapu et al., 2006). pG46 and pG68 plasmids were generously provided by Leonard Wu (Ralf et al., 2006). Nicking enzymes were from New England Biolabs. *E. coli* ssDNA-binding protein was purchased from GE Healthcare.

### ATPase Assays

ATPase assays were performed as described previously, with minor modifications (Johnson et al., 1994). Briefly, the reaction (10 μl) was carried out in buffer A containing 20 mM Tris-HCl (pH 7.0), 1 mM MgCl_2_, 10 mM KCl, 60 μg/ml BSA, 1 mM DTT, and 10% glycerol using 40 nM Rad5, 1 mM ATP trace labeled with [γ-^32^P]ATP, and 0–100 nM DNA as indicated. After incubation at 37°C for 30 min, 1.5 μl of each sample was spotted onto PEI cellulose F thin layer chromatography plate (Merck), followed by resolving the products using a solvent containing 1 M formic acid and 0.25 M LiCl. Products were measured by PhosphorImager (Molecular Dynamics) and quantified by normalizing the measured radioactivity of free phosphate to the total radioactivity in each sample. In each assay set, mixtures without DNA or Rad5 were used as negative controls.

### DNA Helicase Assays

DNA helicase assays were carried out in buffer B containing 20 mM Tris-HCl (pH 7.0), 0.1 mg/ml BSA, 1 mM DTT, and 10% glycerol, which was supplemented with 5 mM of both MgCl_2_ and ATP, except that for assaying the four-way junctions (Figure 2G), 1 mM of both ATP and MgCl_2_ were used, together with 0.5 nM ^32^P-labeled DNA and Rad5 in two-fold increments up to 80 nM (Figures 2B and 2C) or up to 160 nM (Figure 2G), at 25 nM (Figure 2D), or at 120 nM (Figure 2E). After incubating at 37°C for 5 min or for the time indicated on the figure, equal volumes of helicase stop buffer containing 20 mM EDTA, 2 mg/ml Proteinase K, 1% SDS, 10% glycerol, and 0.02% bromophenol blue were added, followed by further incubation for 5 min before loading the DNA samples onto 10% native polyacrylamide gels and separating the products by electrophoresis using 1× Tris-borate buffer containing no EDTA.

Assays with the plasmid-sized forks were carried out essentially as described above but using 5 nM substrate and 80 nM Rad5 unless indicated otherwise. Reactions were quenched after incubation for 5 min at 37°C by using AMP-PNP and small aliquots of the same reaction were tested by restriction enzyme digestion as indicated in Figure 3. We note, however, that excess MgCl_2_ in restriction enzyme buffers alone proved to be sufficient to stop the reaction, which is in agreement with the effect of MgCl_2_ on the rate of branch migration (Panyutin et al., 1995). Products were analyzed by 6% native polyacrylamide electrophoresis using 1× Tris-borate buffer. For the experiments shown in Figure 3D, the activity of *E. coli* single-stranded DNA-binding protein was confirmed in gel shift assay (data not shown). DNA substrates are detailed in the Supplemental Data.

## Supplemental Data

Document S1. Supplemental Results, Supplemental Experimental Procedures, Supplemental References, Two Figures, and One Table
