# Supplementary material for: Yeast Rad5 Protein Required for Postreplication Repair Has a DNA Helicase Activity Specific for Replication Fork Regression
Source: Mol Cell. 2007 Oct 12;28(1):167–75. doi: 10.1016/j.molcel.2007.07.030 (PMC2034406; doi:10.1016/j.molcel.2007.07.030)
Supplement: Document S1. Supplemental Results, Supplemental Experimental Procedures, Supplemental References, Two Figures, and One Table [file mmc1.pdf]

Molecular Cell, Volume *28*

Supplemental Data

Yeast Rad5 Protein Required for Postreplication Repair Has a DNA Helicase Activity Specific for Replication Fork Regression

András Blastyák, Lajos Pintér, Ildiko Unk, Louise Prakash, Satya Prakash, and Lajos Haracska

Supplemental Results

Supplemental Result: Rad5 Binding to DNA Structures

To determine the binding specificity of Rad5 to various DNA structures we carried out gel-shift assays. We found that from a mixture containing equal amounts of radioactively labelled single stranded and partial heteroduplex DNAs, only the partial heteroduplex was bound by Rad5 whereas the amount of free single-stranded DNA did not change (Supplementary Fig. 1A). Next, we checked a variety of DNA structures, including Y-fork, three-way junction, and four-way junction and found that Rad5 was able to bind all these DNAs (Supplementary Fig.1B-D). Since 50% binding of all these DNA substrates occurred at a very similar Rad5 concentration, Rad5 bound these various DNAs with fairly similar affinity. This also indicates that the lack of helicase activity on heteroduplexes and Y-fork is not the consequence of low affinity binding of Rad5 to these substrates.

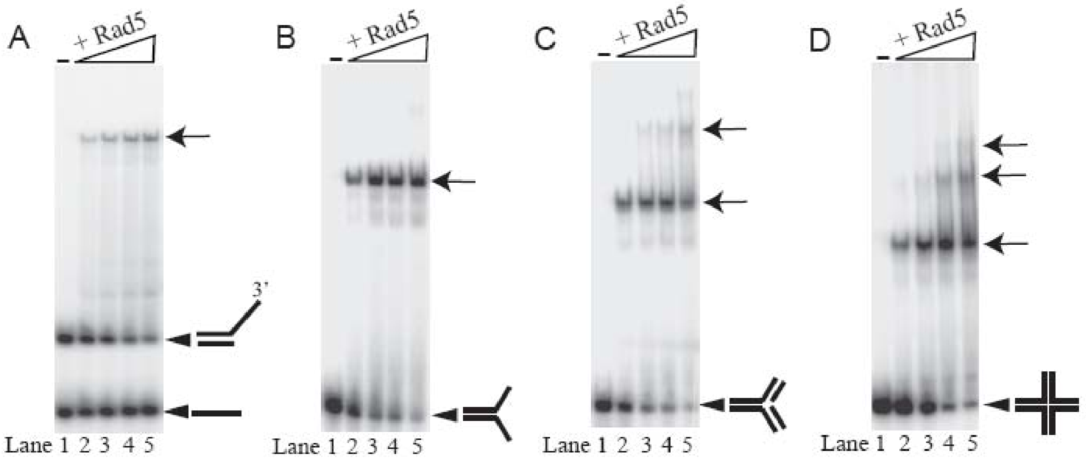

Figure S1. Rad5 Binding to a Variety of DNA Structures

Gel-shift assays were performed in buffer B containing 20 mM Tris-HCl, pH 7.0, 2 mM $MgCl_2$, 0.1 mg/ml BSA, 1mM DTT, and 10% glycerol using 0.5 nM ^{32}P-labeled DNA and Rad5 in two-fold increments up to 70 nM. Reactions were assembled on ice and incubated for 15 min before loading onto a 4% native polyacrylamide gel, containing acrylamide and N,N bis-acrylamide in 30:0.8 ratio, 0.5x Tris-borate, and 2.5% glycerol, followed by electrophoresis at 4°C in 0.5x Tris-borate buffer containing no EDTA. The following DNA substrates were tested: **(A)** a mixture of a 25-nt long single stranded oligonucleotide and a partial heteroduplex obtained by annealing the same 25-nt oligonucleotide to a 50-nt long oligonucleotide; **(B)** Y-fork; **(C)** three-way junction (HetF 25/25); **(D)** four-way junction (X0). The structures of free DNAs are shown schematically and their positions in the gel are indicated by arrowheads while the shifted DNA-Rad5 complexes are shown by arrows. In C and D, DNA-protein complexes suggestive of the binding of more than one Rad5 molecule were also seen.

Supplemental Result: Rad5 Cannot Act as a Canonical DNA Helicase

In order to test if Rad5 could act as a canonical DNA helicase we tested its unwinding activity on short heteroduplexes with either 5’ or 3’ protruding end. Considering the possibility that detecting a weak helicase activity might be hindered by rapid reannealing of the removed strand to its complementary sequence, we also performed these reactions in the presence of E. coli ssDNA binding protein (SSB) that would bind to the released ssDNA thereby preventing reannealing. As shown on Fig. S2A and B, Rad5 did not exhibit any unwinding activity on these substrates. Also, Rad5 was not able to unwind heterologous replication fork-like substrates where only the nascent leading or the nascent lagging strand was present (Fig. S2C and D, respectively). These results are in agreement with the previously reported data (Johnson et al. 1994) and support the notion that Rad5 does not possess canonical DNA helicase activity.

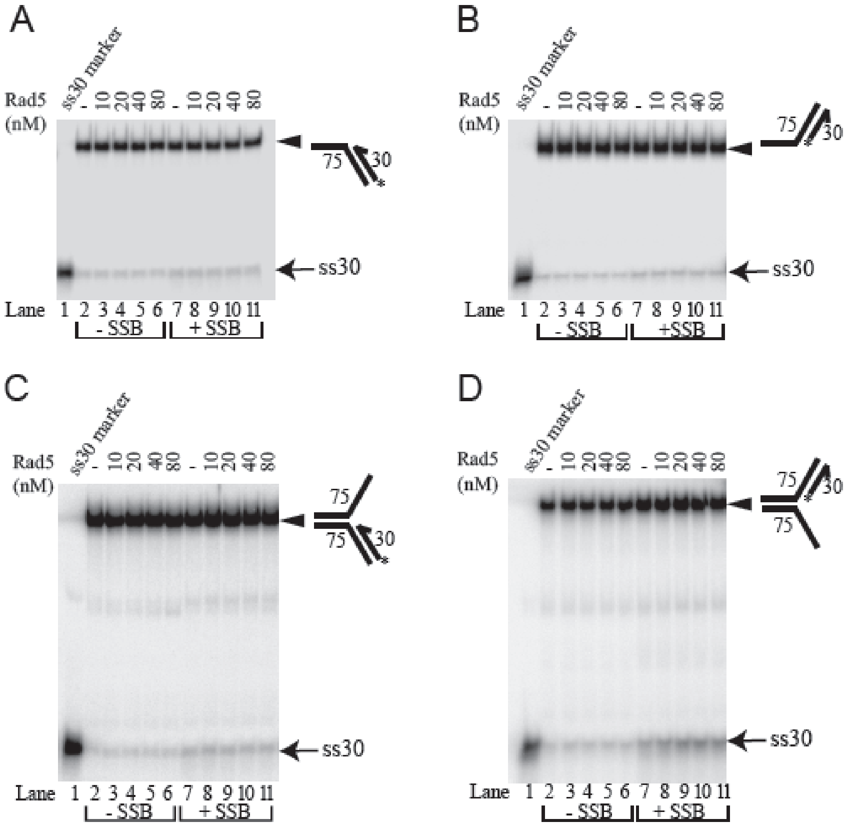

Figure S2. Rad5 Has No Helicase Activity on Heteroduplex DNA and Partial Replication Fork-like Structures

Helicase activity of Rad5 was tested on partial heteroduplex DNAs with 5’ or 3’ overhangs (A and B, respectively) and partial heterologous replication fork-like structures where only the nascent leading or the nascent lagging strand was present (C and D, respectively). Where indicated E. coli SSB was used at 160nM concentration which was added as the last component to the reaction mix followed by 5 minutes incubation at 0 °C before initiating the reaction at 37 °C. The concentration of Rad5, the

structure and the position of the DNA substrate, and the mobility of the ss30 DNA are indicated. The positions of the radioactive labels are marked with asterisks.

Supplemental Experimental Procedures

DNA Substrates

Oligonucleotide-based DNA substrates were generated by annealing oligonucleotides listed in Supplementary Table 1 in various combinations followed by purification from polyacrylamide gel as described (Bachrati and Hickson, 2006). We use the heterologous fork (HetF) term for replication fork-like structures with non-complementary leading and lagging arms, and homologous fork (HomF) term for forks with complementary leading and lagging arms. Oligonucleotides were used in the following combinations, in which we underlined the 5’ ^{32}P-labelled oligonucleotide(s).

Y-fork: O1096/ O1097 (Fig. S1B)

25/50 heteroduplex with 3’ overhang: O1097/ O1099 (Fig. S1A)

30/75 heteroduplex with 5’ overhang: O1054/ O1118 (Fig. S2A)

30/75 heteroduplex with 3’ overhang: O1056/ O1058 (Fig. S2B)

HetF 25/25: O1096/ O1097/ O1098/ O1099 (Fig. S1C)

HetF 30/30: O1054/ O1118/ O1175/ O1176 (Fig. 2B)

HetF 30/0: O1054/ O1175/ O1118 (Fig. S2C)

HetF 0/30: O1176/ O1175/ O1118 (Fig. S2D)

HomF 30/30: O1054/ O1056/ O1058/ O1118 (Fig. 2B, Fig. 2D, and Fig. 2E)

HomF 30/30: O1054/ O1056/ O1058/ O1118 (Fig. 2C)

HomF 30/30: O1054/ O1056/ O1058/ O1118 (Fig. 2F)

The four-way junctions, X0 and X12, used in Fig. 2G and Fig. S1D were made by annealing O1111, O1112, O1113, O1114, and O1114, O1115, O1116, O1117 oligonucleotides, respectively, in which O1114 was 5' ^{32}P-labeled. For experiment shown on Fig. 1D the Y-fork, HetF 25/25, and X12 were used without radioactive label.

The plasmid-sized replication fork model substrate was created essentially as described (Ralf *et al.*, 2006). Briefly, pG46 and pG68 plasmids were gapped by digestion with nicking endonucleases Nt.BbvCI and Nb.BbvCI yielding pG46B and pG68A, respectively. pG46B was then treated with shrimp alkaline phosphatase and subsequently labelled with T4 polynucleotide kinase to yield the plasmid pG46B'. pG68A was linearized with XhoI digestion to yield pG68A Xh. The resulting plasmids containing complementary single stranded gaps were then annealed together at 53°C in 50 mM Tris-HCl pH 7.5, 10 mM $MgCl_2$, 5mM DTT to form a joint molecule. Note, that the joint molecule contains a structural mimic of a stalled replication fork in which the labelled lagging strand is longer by 14 nucleotides. pG68SapI(Het) was created from pG68 by inserting a small sequence heterology by cloning O1301/O1302 heteroduplex oligonucleotides into the SapI site of pG68. To yield pG68ASapI(Het)Xh, pG68SapI(Het) was treated similar to pG68A. We note that in the original description of this experimental system the joint was formed by annealing the two gapped circular plasmids which was subsequently converted to a plectonemic joint by topoisomerase treatment (Ralf *et al.*, 2006). Unlike the RecQ helicases, Rad5 can not dissolve model

forks to parental duplexes and therefore, this conversion was not necessary for our purposes. Thus, we think that the joint configuration shown on Fig. 3A should be considered as a more stringent situation for studying fork reversal than the previously described one.

Table S1. List of Oligonucleotides Used for the Construction of Various DNA Substrates

Oligo name	Length	Sequence	Reference
O1054	30	AgCTACCATgCCTgCCTCAAgAATTCgTAA	This study
O1056	30	TTACgAATTCTTgAggCAggCATggTAgCT	This study
O1058	75	AgCTACCATgCCTgCCTCAAgAATTCgTAATAT gCCTACACTggAgTACCggAgCATCgTCgTgACT gggAAAAC	This study
O1096	50	gTCggATCCTCTAgACAgCTCCATgATCACTgg CACTggTAgAATTCggC	(McGlynn and Lloyd, 2001)
O1097	50	CAACgTCATAgACgATTACATTgCTACATggAg CTgTCTAgAggATCCgA	(McGlynn and Lloyd, 2001)
O1098	26	TgCCgAATTCTACCAgTgCCAgTgAT	(McGlynn and Lloyd, 2001)
O1099	25	TAgCAATgTAATCgTCTATgACgTT	(McGlynn and Lloyd, 2001)
O1111	50	gTCggATCCTCTAgACAgCTCCATgATCACTgg CACTggTAgAATTCggC	(McGlynn *et al.*, 2000)
O1112	50	CAACgTCATAgACgATTACATTgCTACATggAg CTgTCTAgAggATCCgA	(McGlynn *et al.*, 2000)
O1113	50	TgCCgAATTCTACCAgTgCCAgTgATggACATC TTTgCCCACgTTgACCC	(McGlynn *et al.*, 2000)
O1114	50	TgggTCAACgTgggCAAAgATgTCCTAgCAATgT AATCgTCTATgACgTT	(McGlynn *et al.*, 2000)
O1115	49	gACgCTgCCgAATTCTggCTTgCTAggACATCTT TgCCCACgTTgACCC	(McGlynn *et al.*, 2000)

O1116	51	CAACgTCATAgACgATTACATTgCTAggACATgCTgTCTAgAgACTATCgA	(McGlynn *et al.*, 2000)
O1117	50	ATCgATAgTCTCTAgACAgCATgTCCTAgCAAgCCAgAATTCggCAgCgT	(McGlynn *et al.*, 2000)
O1118	75	gTTTTCCCAgTCACgACgATgCTCCggTACTCCAgTgTAggCATATTACgAATTCTTgAggCAggCATggTAgCT	This study
O1175	75	gATCgTTgCATTCATTCTggAggCCTACggTATgCCTACACTggAgTACCggAgCATCgTCgTgACTgggAAAAC	This study
O1176	30	CCgTAggCCTCCAgAATgAATgCAACgATC	This study
O1301	29	gCTCACCTggTAgTCgACTTACgTgATCg	This study
O1302	29	AgCCgATCACgTAAgTCgACTACCAggTg	This study

Supplemental References

Bachrati, C.Z. and Hickson, I.D. (2006). Analysis of the DNA unwinding activity of RecQ family helicases. Methods Enzymol. *409*, 86-100.

Johnson, R.E., Prakash, S., and Prakash, L. (1994). Yeast DNA repair protein RAD5 that promotes instability of simple repetitive sequences is a DNA-dependent ATPase. J. Biol. Chem. *269*, 28259-28262.

McGlynn, P. and Lloyd, R.G. (2001). Rescue of stalled replication forks by RecG: simultaneous translocation on the leading and lagging strand templates supports an active DNA unwinding model of fork reversal and Holliday junction formation. Proc. Natl. Acad. Sci. U. S. A *98*, 8227-8234.

McGlynn, P., Mahdi, A.A., and Lloyd, R.G. (2000). Characterisation of the catalytically active form of RecG helicase. Nucleic Acids Res. *28*, 2324-2332.

Ralf, C., Hickson, I.D., and Wu, L. (2006). The Bloom's syndrome helicase can promote the regression of a model replication fork. J. Biol. Chem. *281*, 22839-22846.
